# Descriptive Epidemiology of Cardiorespiratory Fitness in UK Adults: The Fenland Study

**DOI:** 10.1249/MSS.0000000000003068

**Published:** 2022-10-21

**Authors:** TOMAS I. GONZALES, KATE WESTGATE, STEFANIE HOLLIDGE, TIM LINDSAY, KATRIEN WIJNDAELE, NITA G. FOROUHI, SIMON GRIFFIN, NICK WAREHAM, SOREN BRAGE

**Affiliations:** MRC Epidemiology Unit, University of Cambridge, Cambridge, UNITED KINGDOM

**Keywords:** CARDIORESPIRATORY FITNESS, SOCIODEMOGRAPHIC, PHYSICAL ACTIVITY, ENERGY EXPENDITURE, AGING

## Abstract

**Introduction:**

Cardiorespiratory fitness (CRF) is rarely measured in population studies. Most studies of CRF do not examine differences by population subgroups or seasonal trends. We examined how estimated CRF levels vary by anthropometric, sociodemographic, and behavioral characteristics in a population-based cohort of UK adults (the Fenland Study).

**Methods:**

We used a validated submaximal exercise test to obtain CRF estimates (CRF_estimated_) in 5976 women and 5316 men, residing in the East of England. CRF_estimated_ was defined as estimated maximal oxygen consumption per kilogram total body mass (V̇O_2_max_tbm_) and fat-free mass (V̇O_2_max_ffm_). Descriptive statistics were computed across anthropometric and sociodemographic characteristics, and across the year. Progressive multivariable analyses were performed to examine associations with physical activity energy expenditure (PAEE) and body mass index (BMI).

**Results:**

Mean ± SD V̇O_2_max_tbm_ was lower in women (35.2 ± 7.5 mL·min^−1^·kg^−1^) than men (41.7 ± 7.3 mL·min^−1^·kg^−1^) but V̇O_2_max_ffm_ was similar (women: 59.2 ± 11.6 mL·min^−1^·kg^−1^; men: 62.0 ± 10.3 mL·min^−1^·kg^−1^). CRF_estimated_ was inversely associated with age but not after adjustment for PAEE. People in more physically demanding jobs were fitter compared with those in sedentary jobs, but this association was attenuated in women and reversed in men after adjustment for total PAEE. Physical activity energy expenditure and BMI were positively associated with CRF_estimated_ at all levels of adjustment when expressed relative to fat-free mass. CRF_estimated_ was 4% higher in summer than in winter among women, but did not differ by season among men.

**Conclusions:**

CRF_estimated_ was inversely associated with age but less steeply than anticipated, suggesting older generations are comparatively fitter than younger generations. Physical activity energy expenditure and BMI were stronger determinants of the variance in CRF_estimated_ than other characteristic including age. This emphasizes the importance of modifiable physical activity behaviors in public health interventions.

Cardiorespiratory fitness (CRF) is inversely related to mortality and cardiometabolic disease risk (1–5) but is not widely recognized as a clinical vital sign in the United Kingdom. Most UK primary care providers do not routinely measure CRF, and only a few epidemiological studies have documented CRF levels in UK population subgroups. The Welsh Heart Health Survey (6) and Tuxworth et al. (7) are the earliest epidemiological studies of CRF in UK adults. The Allied-Dunbar National Fitness Survey was the first to establish normative CRF data for the UK population (8). These data were extended by the Northern Ireland Health and Activity Survey (9) and the 2008 Health Survey for England (10). These studies have several strengths: they use dynamic exercise testing to measure differences in CRF levels by anthropometric characteristics, relate CRF to other health behaviors, such as physical activity, and describe differences in CRF by cardiometabolic risk factors. Exercise test selection bias limits generalizability of their findings to the UK population, however, and data on relationships between CRF, sociodemographic characteristics, and clinical characteristics are scarce. It is also unclear how these relationships may be mediated through physical activity. These limitations impede public health action for improved CRF in the population. Here we use a validated submaximal exercise test to obtain CRF estimates (CRF_estimated_) in a population-based cohort of UK adults (the Fenland Study) and examine how CRF_estimated_ levels vary by anthropometric, sociodemographic, and behavioral characteristics.

## METHODS

### Study population

The Fenland Study included 12,435 participants born between 1950 and 1975 and recruited from general practice lists around Cambridgeshire, UK from January 2005 until April 2015, as described in more detail elsewhere (11). Exclusion criteria for participation in the Fenland study were prevalent diabetes, pregnancy or lactation, inability to walk unaided for at least 10 min, psychosis, or terminal illness. Participant eligibility for exercise testing was assessed using a 12-lead resting ECG (Seca CT6i), excluding those presenting with unstable angina. In addition, participants who self-reported having a heart condition were examined by a study nurse to determine whether treadmill testing could be conducted safely. The present analysis included 5976 women and 5316 men with available data on CRF_estimated_. The Health Research Authority NRES Committee East of England-Cambridge Central approved the study in accordance with the Declaration of Helsinki. All participants gave written informed consent. The Fenland Study has a dedicated Patient and Public Involvement panel, who provided input on the acceptability of the study protocols and participant data confidentiality. This study complied with the items listed in the Strengthening the Reporting of Observational Studies in Epidemiology guidelines.

### Anthropometric, sociodemographic, clinical characteristics, and physical activity

Participants arrived at a clinical testing facility to complete baseline assessments after an overnight fast and after abstaining from smoking and vigorous physical activity the morning of their visit. During the clinic visit, height was measured with a rigid stadiometer (SECA 240; Seca, Birmingham, UK), total body mass was measured in light clothing with calibrated scales (TANITA model BC-418 MA; Tanita, Tokyo, Japan), and fat-free mass was measured using dual-energy x-ray absorptiometry (DEXA; Lunar-DPX) (12). Self-report questionnaires were used to determine sociodemographic characteristics, including participant sex, age, ethnicity (White, South Asian, Black, East Asian, other or unknown), education level (basic–compulsory schooling, further–A level (Advanced level subject-based qualification)/apprenticeship/subdegree level, higher-degree level or above), work type (sedentary, standing, manual, retired, unemployed, unknown), annual household income level (<£20,000, £20,000–£40,000, >£40,000), marital status (single, married/living as married, widowed/separated/divorced), smoking status (never, ex-smoker, current), and testing location (Cambridge, Ely, Wisbech). Resting heart rate was measured while supine using a 12-lead ECG (Seca CT6i). After the clinic visit, objective physical activity was assessed using a combined heart rate and uniaxial movement sensor (Actiheart, CamNtech, Cambridge, UK), worn continuously for at least 72 h and at most 6 d. Heart rate was individually calibrated (13) and total physical activity energy expenditure (PAEE) was computed for the wear period as described and validated elsewhere (11,14).

### Cardiorespiratory fitness assessment

We used an incremental, multistage, and submaximal treadmill test to quantify CRF_estimated_, where CRF_estimated_ was defined as estimated maximal oxygen consumption (V̇O_2_max). A diagram and description of the testing procedure is provided in Supplemental Figure 1 (see Supplemental Digital Content, http://links.lww.com/MSS/C731). Heart rate was monitored and recorded during testing using the combined heart rate and movement sensor (Actiheart; CamNtech) (15). The test ended if one of the following criteria were satisfied: 1) leveling-off of heart rate (<3 bpm per min) despite an increase in work rate; 2) reaching 90% of the participant’s age-predicted maximal heart rate (16); 3) exercising above 80% of age-predicted maximal heart rate for over 2 min; 4) reaching a respiratory exchange ratio of 1.1; 5) the participant wanted to stop; 6) participant indication of angina, light-headedness, or nausea; or 7) failure of the testing equipment. For participants on beta blockers (n = 36), the test was terminated after 5 min.

To estimate V̇O_2_max per kg total-body mass (V̇O_2_max_tbm_) from exercise test performance, we extrapolated the linear relationship between heart rate and work rate (17) to age-predicted maximal heart rate (16), which was reduced by 7 bpm in smokers (18,19), added an estimate of resting energy expenditure (20), and then converted the resultant work rate value to net V̇O_2_ using a caloric equivalent for oxygen of 20.35 J·ml O_2_^−1^ (21). The metabolic cost of the treadmill protocol was estimated in an early validation study (13) using the Weir equation to convert oxygen consumption and carbon dioxide production from respiratory gas analysis to energy expenditure, and expressed above measured resting metabolic rate. This approach accounts for anaerobic metabolism and the resulting estimation of activity metabolism for each stage of the treadmill protocol was found to vary minimally between individuals. A separate substudy was conducted to validate the V̇O_2_max estimation approach described above against directly measured V̇O_2_max, demonstrating acceptable agreement (see Supplemental Methods and Results, Supplemental Digital Content, http://links.lww.com/MSS/C731). V̇O_2_max per kg fat-free mass (V̇O_2_max_ffm_) was estimated by multiplying V̇O_2_max_tbm_ values by total body mass and dividing by fat-free mass.

### Statistical analyses

Descriptive statistics were computed across BMI groups and sociodemographic characteristics by sex- and age-stratified groups. Cuzick’s test (22) was performed to test for trend across participant characteristics. Differences in CRF_estimated_ by age, BMI groups (<25, 25 to 30, and >30 kg·m^−2^) and PAEE groups (<40, 40 to 60, and >60 kJ·d^−1^·kg^−1^) were visualized using boxplots. Univariate associations of CRF_estimated_ with age, BMI, and PAEE were computed as Pearson’s *r*; bivariate relationships were investigated using linear regression.

We used sex-stratified and sequentially-adjusted multivariable linear regression to evaluate associations between CRF_estimated_ and sociodemographic characteristics (model 1) with additional adjustment for PAEE (model 2) and BMI (model 3). The season of the year when CRF_estimated_ values were obtained was considered in these analyses by including two orthogonal sine functions in the regression model: “Winter” reaching a value of “1” on January 1 and “−1” on July 1st, and “Spring” reaching a value of “1” on April 1 and “−1” on October 1. Seasonal trends were further described on a monthly basis using a binned regression procedure, controlling for seasonal variation in the measurement of sociodemographic characteristics (23). All analyses were performed in Stata/SE 16.1 (StataCorp, College Station, TX). Statistical significance was set at *P* < 0.05.

## RESULTS

In women, mean ± SD estimated V̇O_2_max_tbm_ was 35.2 ± 7.5 mL O_2_·min^−1^·kg^−1^ and estimated V̇O_2_max_ffm_ was 59.2 ± 11.6 mL O_2_·min^−1^·kg^−1^. In men, estimated V̇O_2_max_tbm_ was 41.7 ± 7.3 mL O_2_·min^−1^·kg^−1^ and estimated V̇O_2_max_ffm_ was 62.0 ± 10.3 mL O_2_·min^−1^·kg^−1^. Per 5 yr age difference, estimated V̇O_2_max_tbm_ was lower on average by 0.2 mL O_2_·min^−1^·kg^−1^ in women and by 0.3 mL O_2_·kg·min^−1^ in men. Estimated V̇O_2_max_ffm_ was not associated with age in women, however in men estimated V̇O_2_max_ffm_ was on average 0.05 mL O_2_·min^−1^·kg^−1^ lower per 5 yr. Trends for other characteristics are reported in Table 1.

**TABLE 1:** Participant characteristics by sex-specific age groups

Sex	Women								
Age Group (yr)	Pooled	29–34	35–39	40–44	45–49	50–54	55–59	60–64	
*N*	5976	184	736	1210	1322	1269	972	283	*P*
Height (cm)	164 ± 6	165 ± 6	165 ± 7	165 ± 6	165 ± 6	164 ± 6	163 ± 6	163 ± 6	<0.01
Total body mass (kg)	70.6 ± 13.9	67.0 ± 12.3	70.0 ± 15.2	71.0 ± 14.7	71.0 ± 13.8	70.8 ± 13.3	70.4 ± 13.1	69.7 ± 12.8	<0.01
Body mass index (kg·m^−2^)	26.2 ± 5.0	24.6 ± 4.2	25.7 ± 5.3	26.2 ± 5.2	26.2 ± 5.0	26.4 ± 4.8	26.4 ± 4.7	26.3 ± 4.6	<0.01
Fat-free mass (kg)	41.4 ± 5.2	40.9 ± 4.5	42.0 ± 5.5	42.1 ± 5.5	41.9 ± 5.2	41.0 ± 5.0	40.3 ± 5.0	39.9 ± 4.9	<0.01
Resting heart rate (bpm)	64 ± 8	64 ± 8	64 ± 9	64 ± 89	65 ± 9	64 ± 8	64 ± 8	63 ± 8	0.08
V̇O_2_max per kg bodyweight (mL O_2_·min^−1^·kg^−1^)	35.2 ± 7.5	35.9 ± 6.2	36.0 ± 7.0	35.5 ± 7.1	35.3 ± 7.5	34.8 ± 7.6	34.6 ± 7.9	34.6 ± 8.3	<0.01
V̇O_2_max per kg fat-free mass (mL O_2_·min^−1^·kg^−1^)	59.2 ± 11.6	58.1 ± 8.8	58.8 ± 10.1	58.8 ± 10.3	59.0 ± 11.4	59.5 ± 12.4	59.8 ± 13.0	59.7 ± 13.8	0.12
PAEE (kJ·d^−1^·kg^−1^)	51 ± 20	59 ± 21	57 ± 21	53 ± 20	51 ± 20	48 ± 19	46 ± 19	43 ± 16	<0.01

Sex	Men								
Age Group (yr)	Pooled	29–34	35–39	40–44	45–49	50–54	55–59	60–64	
*N*	5316	161	735	1010	1135	1072	929	274	*P*
Height (cm)	178 ± 7	179 ± 7	178 ± 7	179 ± 7	178 ± 7	177 ± 7	176 ± 7	177 ± 7	<0.01
Bodyweight (kg)	85.9 ± 13.9	84.6 ± 13.6	84.7 ± 14.6	86.2 ± 13.9	86.1 ± 13.9	86.4 ± 13.3	85.8 ± 13.9	85.4 ± 14.2	0.03
Body mass index (kg·m^−2^)	27.2 ± 4.0	26.4 ± 3.8	26.7 ± 4.2	27.0 ± 3.9	27.2 ± 4.0	27.5 ± 4.0	27.5 ± 4.0	27.3 ± 3.9	<0.01
Fat-free mass (kg)	57.3 ± 6.8	57.6 ± 6.8	57.4 ± 7.0	58.1 ± 6.7	57.6 ± 6.7	57.3 ± 6.7	56.3 ± 6.7	56.3 ± 7.0	<0.01
Resting heart rate (bpm)	61 ± 9	62 ± 9	60 ± 9	61 ± 9	61 ± 9	61 ± 9	62 ± 9	62 ± 9	<0.01
V̇O_2_max per kg bodyweight (mL O_2_·min^−1^·kg^−1^)	41.7 ± 7.3	42.0 ± 6.0	42.9 ± 6.9	42.6 ± 6.9	42.0 ± 7.3	41.0 ± 7.0	40.9 ± 8.0	40.4 ± 8.9	<0.01
V̇O_2_max per kg fat-free mass (mL O_2_·min^−1^·kg^−1^)	62.0 ± 10.3	61.0 ± 8.4	62.5 ± 9.5	62.6 ± 9.3	62.4 ± 10.4	61.4 ± 10.1	61.6 ± 11.3	60.8 ± 13.0	<0.01
PAEE (kJ·d^−1^·kg^−1^)	59 ± 23	65 ± 25	66 ± 24	64 ± 24	60 ± 23	57 ± 22	52 ± 21	47 ± 21	<0.01

The Fenland study 2005 to 2015.

Values are mean ± SD, unless otherwise indicated. *P* value computed from Cuzick’s test for trend.

**FIGURE 1:**
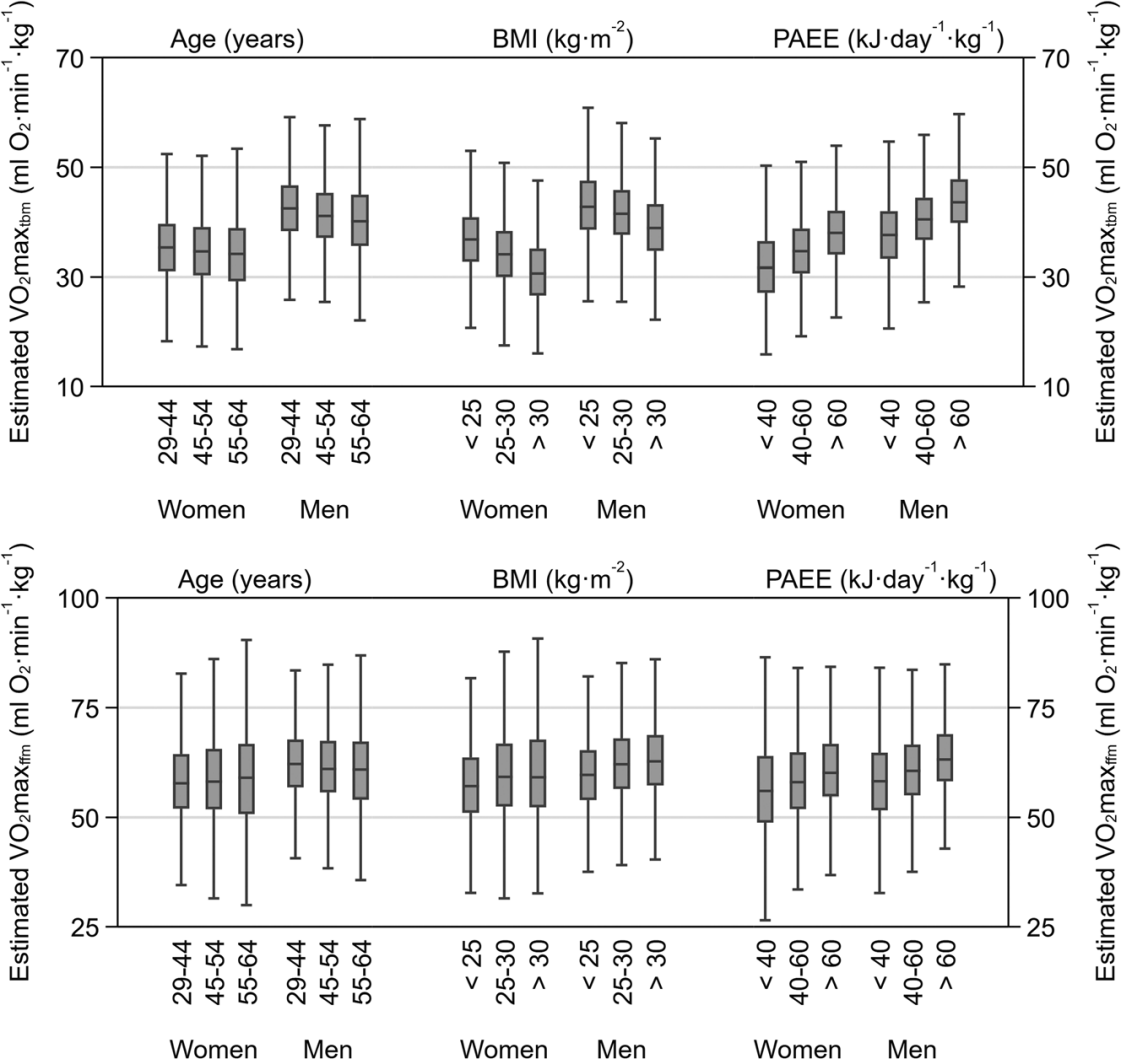
Sex-stratified estimated maximal oxygen consumption per kilogram total body mass (V̇O_2_max_tbm_; *top panel*) and per kilogram fat-free mass (V̇O_2_max_ffm_; *bottom panel*) by age, BMI, and PAEE. *Box plots* represent medians, interquartile ranges, and minimum–maximum ranges without outliers. The Fenland Study 2005 to 2015.

Figure 1 shows differences in estimated V̇O_2_max_tbm_ and V̇O_2_max_ffm_ by age, BMI groups, and PAEE groups. The association of CRF_estimated_ with age, BMI, and PAEE was strongest when expressed as V̇O_2_max_tbm_ compared with V̇O_2_max_ffm_. We investigated this further by conducting univariate and bivariate analyses of estimated V̇O_2_max_tbm_ and V̇O_2_max_ffm_ with BMI, PAEE, and age (Supplemental Figs. 2 and 3, Supplemental Digital Content, http://links.lww.com/MSS/C731). The association of estimated V̇O_2_max_tbm_ with PAEE (Pearson’s *r* for women: 0.38; men: *r*: 0.38) was higher than associations with BMI (Pearson’s r for women: −0.34; men: −0.24) and age (Pearson’s *r* for women: −0.06; men: −0.11). The combination of BMI and PAEE explained more variance in estimated V̇O_2_max_tbm_ (20% for women, 17% for men) than bivariate combinations with age. For estimated V̇O_2_max_ffm_, univariate and bivariate analyses had weaker associations and less explained variance than analogous results for estimated V̇O_2_max_tbm_.

Unadjusted mean V̇O_2_max_tbm_ estimates, stratified by sex and sociodemographic characteristics, are provided in Table 2. Estimated V̇O_2_max_tbm_ was generally higher in participants with higher educational attainment (Women: higher education: 36.0 ± 7.2 mL O_2_·min^−1^·kg^−1^; no education: 33.0 ± 6.9 mL O_2_·min^−1^·kg^−1^; men: higher education: 42.0 ± 7.1 mL O_2_·min^−1^·kg^−1^; no education: 40.3 ± 6.7 mL O_2_·min^−1^·kg^−1^). Workers in more physically demanding jobs were fitter than those in sedentary jobs (female manual workers: 35.9 ± 8.0 mL O_2_·min^−1^·kg^−1^; female sedentary workers: 34.9 ± 7.0 mL O_2_·min^−1^·kg^−1^; male manual workers: 42.4 ± 7.8 mL O_2_·min^−1^·kg^−1^ male sedentary workers: 41.7 ± 6.9 mL O_2_·min^−1^·kg^−1^). Current smokers had similar V̇O_2_max_tbm_ estimates compared with nonsmokers (female smokers: 35.7 ± 8.0 mL O_2_·min^−1^·kg^−1^; female nonsmokers: 35.0 ± 7.4 mL O_2_·min^−1^·kg^−1^; male smokers: 41.7 ± 7.7 mL O_2_·min^−1^·kg^−1^; male nonsmokers: 41.8 ± 7.2 mL O_2_·min^−1^·kg^−1^). Estimated V̇O_2_max_tbm_ differed by ethnicity; however, sample sizes were disproportionate between Whites and other racial and ethnic groups. Differences in estimated V̇O_2_max_tbm_ were not apparent between income levels, marital status, and testing sites. Analogous results for estimated V̇O_2_max_ffm_ are presented in Table 3.

**TABLE 2:** Sequentially adjusted multivariable analysis of estimated V̇O_2_max per kg total body mass (mL O_2_·min^−1^·kg^−1^) by sociodemographic characteristics.

Sex (*N*)	Women (5784)					Men (5209)				
Model	Count (%)	Unadjusted	Model 1	Model 2	Model 3	Count (%)	Unadjusted	Model 1	Model 2	Model 3
Reference mean			35.6* (34.3; 37.0)	35.2* (34.0; 36.5)	34.7* (33.5; 36.0)			42.3* (40.9; 43.7)	42.2* (40.9; 43.6)	41.8* (40.5; 43.1)
Age group (yr)										
29–34	171 (3.0%)	35.9 ± 6.2	Reference	Reference	Reference	154 (3.0%)	42.0 ± 6.2	Reference	Reference	Reference
35–39	711 (12.3%)	36.0 ± 7.0	−0.6 (−1.9; 0.6)	−0.4 (−1.6; 0.7)	−0.1 (−1.2; 1.0)	714 (13.7%)	43.0 ± 6.9	−0.0 (−1.3; 1.3)	−0.1 (−1.3; 1.1)	−0.0 (−1.2; 1.1)
40–44	1168 (20.2%)	35.5 ± 7.1	−1.1 (−2.3; 0.1)	−0.3 (−1.5; 0.8)	0.0 (−1.1; 1.1)	994 (19.1%)	42.7 ± 6.9	−0.5 (−1.8; 0.7)	−0.2 (−1.4; 0.9)	−0.1 (−1.3; 1.0)
45–49	1278 (22.1%)	35.3 ± 7.5	−1.3** (−2.5; −0.1)	−0.2 (−1.4; 0.9)	0.0 (−1.1; 1.1)	1107 (21.3%)	42.2 ± 7.2	−1.2 (−2.4; 0.1)	−0.3 (−1.5; 0.8)	−0.2 (−1.4; 0.9)
50–54	1229 (21.2%)	34.8 ± 7.6	−1.8* (−3.0; −0.6)	−0.3 (−1.5; 0.8)	0.0 (−1.1; 1.0)	1052 (20.2%)	41.1 ± 7.0	−2.1* (−3.4; −0.9)	−0.8 (−2.0; 0.3)	−0.7 (−1.8; 0.5)
55–59	946 (16.4%)	34.6 ± 7.9	−2.2* (−3.4; −0.9)	−0.4 (−1.5; 0.8)	−0.2 (−1.3; 1.0)	917 (17.6%)	40.8 ± 7.9	−2.5* (−3.7; −1.2)	−0.6 (−1.8; 0.6)	−0.5 (−1.7; 0.7)
60–64	281 (4.9%)	34.6 ± 8.3	−2.4* (−3.9; −0.9)	−0.2 (−1.6; 1.2)	−0.1 (−1.5; 1.3)	271 (5.2%)	40.4 ± 8.8	−3.1* (−4.6; −1.6)	−0.4 (−1.8; 1.0)	−0.4 (−1.8; 0.9)
Ethnicity										
White	5357 (92.6%)	35.3 ± 7.4	Reference	Reference	Reference	4816 (92.5%)	42.0 ± 7.3	Reference	Reference	Reference
South Asian	63 (1.1%)	34.7 ± 10.5	−1.1 (−3.0; 0.7)	−0.1 (−1.8; 1.6)	−0.2 (−1.8; 1.5)	70 (1.3%)	40.3 ± 7.3	−1.4 (−3.1; 0.3)	−0.5 (−2.1; 1.1)	−0.9 (−2.5; 0.6)
Black	21 (0.4%)	34.8 ± 8.0	−1.4 (−4.5; 1.8)	−1.4 (−4.4; 1.5)	−0.8 (−3.6; 2.1)	28 (0.5%)	38.6 ± 8.2	−3.2** (−5.8; −0.5)	−2.1 (−4.6; 0.4)	−1.9 (−4.4; 0.5)
East Asian	38 (0.7%)	38.7 ± 9.8	2.9** (0.5; 5.3)	3.7* (1.5; 5.9)	2.8** (0.7; 4.9)	19 (0.4%)	43.5 ± 8.7	1.8 (−1.5; 5.1)	2.6 (−0.5; 5.6)	1.8 (−1.2; 4.8)
Others or unknown	305 (5.3%)	32.5 ± 6.6	−2.1* (−3.1; −1.2)	−1.6* (−2.4; −0.7)	−1.9* (−2.7; −1.1)	276 (5.3%)	39.5 ± 6.0	−2.2* (−3.2; −1.2)	−1.7* (−2.6; −0.7)	−1.7* (−2.6; −0.9)
Education level										
None	31 (0.5%)	33.0 ± 6.9	−1.6 (−4.3; 1.0)	−1.4 (−3.9; 1.0)	−1.5 (−3.9; 0.9)	21 (0.4%)	40.3 ± 6.7	−0.3 (−3.4; 2.9)	0.3 (−2.6; 3.2)	0.5 (−2.4; 3.4)
Basic	1191 (20.6%)	34.7 ± 8.0	Reference	Reference	Reference	859 (16.5%)	42.0 ± 7.8	Reference	Reference	Reference
Further	2617 (45.2%)	34.8 ± 7.4	0.1 (−0.4; 0.6)	−0.1 (−0.6; 0.4)	−0.2 (−0.7; 0.3)	2426 (46.6%)	41.6 ± 7.3	−0.2 (−0.8; 0.3)	−0.1 (−0.6; 0.4)	−0.1 (−0.6; 0.4)
Higher	1945 (33.6%)	36.0 ± 7.2	1.1* (0.5; 1.7)	0.7** (0.2; 1.3)	0.2 (−0.3; 0.7)	1903 (36.5%)	42.0 ± 7.1	0.4 (−0.3; 1.1)	0.5 (−0.1; 1.1)	0.1 (−0.5; 0.8)
Work type										
Sedentary	2762 (47.8%)	34.9 ± 7.0	Reference	Reference	Reference	2721 (52.2%)	41.7 ± 6.9	Reference	Reference	Reference
Standing	1852 (32.0%)	35.5 ± 7.7	0.9* (0.4; 1.3)	−0.1 (−0.5; 0.3)	−0.0 (−0.4; 0.4)	703 (13.5%)	42.0 ± 7.6	0.9* (0.2; 1.5)	−0.3 (−0.8; 0.3)	−0.1 (−0.7; 0.4)
Manual	437 (7.6%)	35.9 ± 8.0	1.6* (0.8; 2.4)	−0.4 (−1.1; 0.4)	−0.0 (−0.7; 0.7)	1517 (29.1%)	42.4 ± 7.8	1.2* (0.6; 1.7)	−1.0* (−1.5; −0.5)	−0.9* (−1.4; −0.4)
Retired	160 (2.8%)	34.3 ± 7.5	0.3 (−1.0; 1.6)	−0.3 (−1.5; 0.9)	−0.2 (−1.3; 1.0)	112 (2.2%)	39.8 ± 7.2	−0.5 (−2.0; 0.9)	−1.5** (−2.8; −0.2)	−1.4** (−2.7; −0.1)
Unemployed	61 (1.1%)	34.6 ± 7.8	−0.7 (−2.5; 1.2)	−0.6 (−2.3; 1.1)	0.1 (−1.6; 1.8)	64 (1.2%)	39.3 ± 6.8	−1.8 (−3.6; 0.1)	−2.2** (−3.9; −0.5)	−1.9** (−3.6; −0.3)
Unknown	512 (8.9%)	35.4 ± 8.2	0.7** (0.0; 1.4)	−0.1 (−0.7; 0.6)	−0.1 (−0.8; 0.5)	92 (1.8%)	40.7 ± 9.2	0.3 (−1.3; 1.8)	0.6 (−0.9; 2.0)	0.8 (−0.6; 2.2)
Income										
< £20,000	892 (15.4%)	34.8 ± 7.8	−0.2 (−0.8; 0.5)	0.2 (−0.3; 0.8)	0.2 (−0.3; 0.8)	472 (9.1%)	41.4 ± 7.9	0.2 (−0.6; 0.9)	0.8** (0.0; 1.5)	0.6 (−0.1; 1.3)
£20,000–£40,000	2038 (35.2%)	34.9 ± 7.5	Reference	Reference	Reference	1719 (33.0%)	41.5 ± 7.8	Reference	Reference	Reference
> £40,000	2691 (46.5%)	35.5 ± 7.2	0.4 (−0.0; 0.9)	0.1 (−0.3; 0.5)	0.1 (−0.3; 0.5)	2928 (56.2%)	42.0 ± 6.9	0.5** (0.1; 1.0)	0.3 (−0.1; 0.8)	0.4 (−0.0; 0.8)
Unknown	163 (2.8%)	35.5 ± 9.9	1.1 (−0.1; 2.3)	0.9 (−0.2; 2.0)	0.9 (−0.2; 2.0)	90 (1.7%)	42.4 ± 7.6	0.7 (−0.8; 2.3)	1.5** (0.0; 2.9)	1.3 (−0.1; 2.8)
Marital status										
Single	380 (6.6%)	35.9 ± 7.1	0.3 (−0.6; 1.1)	0.2 (−0.6; 0.9)	0.3 (−0.5; 1.0)	389 (7.5%)	42.2 ± 7.8	−0.1 (−0.9; 0.7)	0.2 (−0.5; 1.0)	0.0 (−0.7; 0.7)
Married/living as married	3547 (61.3%)	35.5 ± 7.5	Reference	Reference	Reference	3350 (64.3%)	42.0 ± 7.4	Reference	Reference	Reference
Widowed/separated/divorced	489 (8.5%)	35.7 ± 7.7	0.5 (−0.2; 1.3)	0.4 (−0.3; 1.0)	0.4 (−0.3; 1.0)	277 (5.3%)	42.3 ± 7.2	0.6 (−0.3; 1.5)	0.4 (−0.4; 1.2)	0.4 (−0.5; 1.2)
Unknown	1368 (23.7%)	33.9 ± 7.1	−1.4* (−1.9; −0.9)	−1.4* (−1.9; −0.9)	−1.3* (−1.8; −0.9)	1193 (22.9%)	40.8 ± 6.7	−1.4* (−2.0; −0.8)	−1.3* (−1.8; −0.8)	−1.4* (−1.9; −0.8)
Smoker status										
Never smoked	3246 (56.1%)	35.0 ± 7.4	Reference	Reference	Reference	2718 (52.2%)	41.8 ± 7.2	Reference	Reference	Reference
Ex-smoker	1858 (32.1%)	35.3 ± 7.4	0.4 (−0.0; 0.8)	0.1 (−0.3; 0.4)	0.3 (−0.1; 0.7)	1760 (33.8%)	41.9 ± 7.4	0.3 (−0.2; 0.7)	0.1 (−0.4; 0.5)	0.2 (−0.2; 0.6)
Current smoker	615 (10.6%)	35.7 ± 8.0	1.0* (0.4; 1.7)	−0.4 (−1.0; 0.3)	−0.1 (−0.7; 0.5)	680 (13.1%)	41.7 ± 7.7	−0.1 (−0.7; 0.6)	−1.3* (−1.9; −0.7)	−1.3* (−1.9; −0.7)
Unknown	65 (1.1%)	34.7 ± 6.3	−0.1 (−1.9; 1.7)	−0.8 (−2.5; 0.9)	−0.6 (−2.2; 1.0)	51 (1.0%)	40.8 ± 7.2	−0.9 (−2.9; 1.1)	−1.4 (−3.2; 0.5)	−1.1 (−2.9; 0.8)
Test site										
Cambridge	2060 (35.6%)	35.6 ± 7.5	Reference	Reference	Reference	1995 (38.3%)	41.7 ± 7.5	Reference	Reference	Reference
Ely	2201 (38.1%)	35.1 ± 7.4	0.3 (−0.2; 0.8)	0.6** (0.1; 1.0)	0.8* (0.4; 1.2)	1841 (35.3%)	41.9 ± 7.2	0.8* (0.3; 1.3)	0.8* (0.4; 1.3)	1.2* (0.7; 1.6)
Wisbech	1523 (26.3%)	34.7 ± 7.4	−0.3 (−0.8; 0.3)	−0.0 (−0.5; 0.5)	0.4 (−0.1; 0.9)	1373 (26.4%)	41.9 ± 7.3	0.4 (−0.2; 1.0)	0.3 (−0.2; 0.8)	0.8* (0.3; 1.4)
Seasonality										
Spring			0.0 (−0.3; 0.3)	0.1 (−0.2; 0.3)	0.1 (−0.1; 0.3)			−0.2 (−0.4; 0.1)	−0.1 (−0.3; 0.2)	−0.1 (−0.4; 0.2)
Winter			−0.7* (−1.0; −0.4)	−0.4* (−0.7; −0.2)	−0.5* (−0.7; −0.2)			−0.4* (−0.7; −0.1)	0.0 (−0.3; 0.2)	0.0 (−0.3; 0.3)
PAEE (kJ·d^−1^·kg^−1^)				0.1* (0.1; 0.2)	0.1* (0.1; 0.1)				0.1* (0.1; 0.1)	0.1* (0.1; 0.1)
BMI (kg·m^−2^)					−0.4* (−0.4; −0.3)					−0.3* (−0.4; −0.3)

The Fenland study 2005 to 2015.

Unadjusted values represent mean ± SD V̇O_2_max estimates computed within each substratum. Model values are mean (95% CI). Model 1 is mutually adjusted for sociodemographic characteristics. Model 2 is additionally adjusted for PAEE (kJ·d^−1^·kg^−1^). Model 3 is additionally adjusted for BMI (kg·m^−2^). Mean ± SD PAEE for women: 49 ± 20 kJ·d^−1^·kg^−1^; for men: 59 ± 23 kJ·d^−1^·kg^−1^. Mean ± SD BMI for women: 26.5 ± 5.3 kg·m^−2^; for men: 27.3 ± 4.1 kg·m^−2^.

*P < 0.01.

**P < 0.05.

**TABLE 3:** Sequentially adjusted multivariable analysis of estimated maximal oxygen consumption (V̇O_2_max) per kg fat free mass (mL O_2_·min^−1^·kg^−1^) by sociodemographic characteristics.

Sex (*N*)	Women (5530)					Men (4980)				
Model	Count (%)	Unadjusted	Model 1	Model 2	Model 3	Count (%)	Unadjusted	Model 1	Model 2	Model 3
Reference mean			59.3* (57.1; 61.5)	59.1* (56.9; 61.3)	59.5* (57.4; 61.7)			61.7* (59.5; 63.8)	61.7* (59.6; 63.7)	62.3* (60.2; 64.3)
Age group (yr)										
29–34	157 (2.8%)	58.1 ± 8.8	Reference	Reference	Reference	136 (2.7%)	61.0 ± 8.4	Reference	Reference	Reference
35–39	664 (12.0%)	58.8 ± 10.1	−0.7 (−2.8; 1.3)	−0.6 (−2.6; 1.4)	−0.9 (−2.8; 1.1)	673 (13.5%)	62.5 ± 9.5	0.3 (−1.6; 2.3)	0.2 (−1.6; 2.1)	0.1 (−1.7; 1.9)
40–44	1104 (20.0%)	58.8 ± 10.3	−0.9 (−2.9; 1.1)	−0.4 (−2.3; 1.6)	−0.7 (−2.6; 1.2)	950 (19.1%)	62.6 ± 9.3	0.1 (−1.8; 2.0)	0.3 (−1.5; 2.2)	0.2 (−1.6; 2.0)
45–49	1224 (22.1%)	59.0 ± 11.4	−0.7 (−2.7; 1.3)	0.0 (−1.9; 2.0)	−0.3 (−2.2; 1.7)	1053 (21.1%)	62.4 ± 10.4	−0.2 (−2.1; 1.7)	0.4 (−1.4; 2.3)	0.3 (−1.5; 2.1)
50–54	1179 (21.3%)	59.5 ± 12.4	−0.2 (−2.2; 1.8)	0.8 (−1.2; 2.7)	0.5 (−1.4; 2.4)	1006 (20.2%)	61.4 ± 10.1	−1.2 (−3.0; 0.7)	−0.1 (−1.9; 1.8)	−0.3 (−2.1; 1.5)
55–59	921 (16.7%)	59.8 ± 13.0	−0.3 (−2.4; 1.7)	0.9 (−1.1; 3.0)	0.7 (−1.2; 2.7)	892 (17.9%)	61.6 ± 11.3	−1.3 (−3.3; 0.6)	0.2 (−1.7; 2.1)	0.0 (−1.8; 1.9)
60–64	281 (5.1%)	59.7 ± 13.8	−0.7 (−3.2; 1.7)	0.8 (−1.6; 3.2)	0.7 (−1.7; 3.0)	270 (5.4%)	60.8 ± 13.0	−2.1 (−4.3; 0.1)	0.1 (−2.1; 2.3)	0.1 (−2.1; 2.2)
Ethnicity										
White	5144 (93.0%)	59.4 ± 11.6	Reference	Reference	Reference	4634 (93.1%)	62.2 ± 10.3	Reference	Reference	Reference
South Asian	61 (1.1%)	60.2 ± 15.6	1.4 (−1.6; 4.3)	2.1 (−0.8; 5.0)	2.1 (−0.8; 5.0)	70 (1.4%)	61.8 ± 11.6	0.2 (−2.2; 2.7)	1.0 (−1.4; 3.3)	1.5 (−0.8; 3.8)
Black	21 (0.4%)	56.4 ± 13.1	−2.9 (−7.9; 2.0)	−2.9 (−7.8; 1.9)	−3.6 (−8.4; 1.3)	26 (0.5%)	54.5 ± 11.7	−7.5* (−11.5; −3.6)	−6.8* (−10.7; −3.0)	−7.1* (−10.9; −3.3)
East Asian	38 (0.7%)	61.8 ± 14.4	3.0 (−0.7; 6.7)	3.5 (−0.1; 7.2)	4.4** (0.8; 8.0)	19 (0.4%)	62.3 ± 12.5	1.3 (−3.4; 5.9)	1.9 (−2.6; 6.4)	2.9 (−1.5; 7.3)
Others or unknown	266 (4.8%)	54.2 ± 10.0	−3.5* (−5.1; −2.0)	−3.2* (−4.8; −1.7)	−2.9* (−4.5; −1.4)	231 (4.6%)	58.7 ± 8.2	−2.4* (−3.9; −0.9)	−1.9** (−3.4; −0.4)	−1.9** (−3.3; −0.4)
Education level										
None	28 (0.5%)	57.6 ± 13.7	−2.7 (−7.0; 1.7)	−2.8 (−7.0; 1.5)	−2.9 (−7.1; 1.4)	18 (0.4%)	62.6 ± 11.0	−0.2 (−5.0; 4.6)	0.8 (−3.9; 5.4)	0.4 (−4.2; 5.0)
Basic	1113 (20.1%)	59.9 ± 13.0	Reference	Reference	Reference	790 (15.9%)	62.9 ± 10.8	Reference	Reference	Reference
Further	2486 (45.0%)	59.1 ± 11.6	−0.5 (−1.3; 0.4)	−0.6 (−1.4; 0.2)	−0.5 (−1.3; 0.3)	2295 (46.1%)	62.2 ± 10.5	−0.4 (−1.3; 0.4)	−0.4 (−1.2; 0.4)	−0.4 (−1.3; 0.4)
Higher	1903 (34.4%)	58.9 ± 10.8	−0.5 (−1.5; 0.4)	−0.9 (−1.8; 0.1)	−0.4 (−1.3; 0.6)	1877 (37.7%)	61.4 ± 9.8	−0.8 (−1.8; 0.2)	−0.7 (−1.7; 0.2)	−0.3 (−1.3; 0.6)
Work type										
Sedentary	2665 (48.2%)	58.7 ± 10.8	Reference	Reference	Reference	2639 (53.0%)	61.7 ± 9.7	Reference	Reference	Reference
Standing	1763 (31.9%)	59.7 ± 12.1	0.8** (0.1; 1.5)	0.1 (−0.6; 0.8)	−0.0 (−0.7; 0.7)	665 (13.4%)	62.7 ± 10.9	0.9 (−0.0; 1.8)	−0.1 (−0.9; 0.8)	−0.2 (−1.1; 0.6)
Manual	407 (7.4%)	60.4 ± 13.5	1.6* (0.4; 2.9)	0.3 (−0.9; 1.5)	−0.0 (−1.2; 1.2)	1419 (28.5%)	62.5 ± 10.8	0.3 (−0.4; 1.1)	−1.6* (−2.3; −0.8)	−1.7* (−2.5; −1.0)
Retired	159 (2.9%)	59.8 ± 13.2	0.7 (−1.3; 2.7)	0.2 (−1.8; 2.2)	0.0 (−1.9; 2.0)	111 (2.2%)	59.6 ± 10.4	−1.6 (−3.6; 0.4)	−2.4** (−4.4; −0.4)	−2.5** (−4.4; −0.5)
Unemployed	58 (1.0%)	59.8 ± 12.0	0.6 (−2.4; 3.6)	0.6 (−2.4; 3.6)	−0.0 (−3.0; 2.9)	60 (1.2%)	59.3 ± 9.3	−2.9** (−5.6; −0.3)	−3.1** (−5.7; −0.5)	−3.7* (−6.2; −1.1)
Unknown	478 (8.6%)	58.9 ± 11.8	0.1 (−1.0; 1.3)	−0.5 (−1.6; 0.7)	−0.4 (−1.5; 0.7)	86 (1.7%)	61.4 ± 14.4	−0.3 (−2.6; 2.0)	0.1 (−2.1; 2.3)	−0.2 (−2.4; 1.9)
Income										
< £20,000	838 (15.2%)	59.7 ± 12.6	0.2 (−0.7; 1.2)	0.5 (−0.4; 1.5)	0.6 (−0.4; 1.5)	430 (8.6%)	62.0 ± 11.8	0.6 (−0.5; 1.8)	1.1** (0.0; 2.2)	1.3** (0.2; 2.4)
£20,000–£40,000	1924 (34.8%)	59.2 ± 11.7	Reference	Reference	Reference	1620 (32.5%)	61.7 ± 10.9	Reference	Reference	Reference
> £40,000	2617 (47.3%)	58.9 ± 10.9	0.1 (−0.6; 0.8)	−0.1 (−0.8; 0.6)	−0.0 (−0.8; 0.7)	2850 (57.2%)	62.1 ± 9.6	0.8** (0.1; 1.5)	0.6 (−0.1; 1.3)	0.5 (−0.2; 1.2)
Unknown	151 (2.7%)	60.6 ± 15.9	1.4 (−0.5; 3.3)	1.2 (−0.7; 3.1)	1.2 (−0.7; 3.1)	80 (1.6%)	63.3 ± 11.9	1.6 (−0.7; 4.0)	2.4** (0.1; 4.7)	2.6** (0.3; 4.8)
Marital status										
Single	377 (6.8%)	60.2 ± 11.5	0.7 (−0.6; 2.0)	0.6 (−0.7; 1.9)	0.5 (−0.7; 1.8)	383 (7.7%)	61.7 ± 11.4	−0.4 (−1.5; 0.8)	−0.2 (−1.3; 0.9)	0.2 (−0.9; 1.3)
Married/living as married	3519 (63.6%)	59.6 ± 11.8	Reference	Reference	Reference	3328 (66.8%)	62.4 ± 10.4	Reference	Reference	Reference
Widowed/separated/divorced	486 (8.8%)	60.1 ± 12.0	0.5 (−0.7; 1.6)	0.3 (−0.8; 1.5)	0.3 (−0.9; 1.4)	275 (5.5%)	62.6 ± 10.4	0.4 (−0.9; 1.7)	0.2 (−1.0; 1.4)	0.2 (−1.0; 1.5)
Unknown	1148 (20.8%)	57.1 ± 10.8	−2.1* (−2.9; −1.2)	−2.1* (−2.9; −1.2)	−2.1* (−3.0; −1.3)	994 (20.0%)	60.7 ± 9.4	−1.6* (−2.5; −0.8)	−1.6* (−2.4; −0.8)	−1.5* (−2.3; −0.7)
Smoker status										
Never smoked	3126 (56.5%)	58.8 ± 11.5	Reference	Reference	Reference	2608 (52.4%)	61.7 ± 10.0	Reference	Reference	Reference
Ex-smoker	1770 (32.0%)	59.6 ± 11.7	0.7** (0.1; 1.4)	0.5 (−0.2; 1.2)	0.3 (−0.4; 0.9)	1695 (34.0%)	62.8 ± 10.5	1.2* (0.5; 1.8)	1.0* (0.4; 1.6)	0.7** (0.1; 1.3)
Current smoker	573 (10.4%)	59.7 ± 12.2	0.7 (−0.3; 1.8)	−0.3 (−1.3; 0.7)	−0.5 (−1.5; 0.5)	628 (12.6%)	61.3 ± 10.9	−0.4 (−1.4; 0.5)	−1.5* (−2.4; −0.6)	−1.5* (−2.4; −0.6)
Unknown	61 (1.1%)	58.5 ± 11.0	−0.1 (−3.0; 2.8)	−0.6 (−3.5; 2.3)	−1.0 (−3.8; 1.9)	49 (1.0%)	60.3 ± 9.2	−1.0 (−3.9; 1.9)	−1.6 (−4.4; 1.2)	−1.8 (−4.5; 1.0)
Test site										
Cambridge	2044 (37.0%)	59.4 ± 11.6	Reference	Reference	Reference	1982 (39.8%)	61.7 ± 10.6	Reference	Reference	Reference
Ely	2190 (39.6%)	59.1 ± 12.0	0.5 (−0.2; 1.3)	0.7 (−0.0; 1.5)	0.5 (−0.2; 1.3)	1833 (36.8%)	62.4 ± 10.0	1.1* (0.4; 1.8)	1.2* (0.5; 1.9)	0.7** (0.0; 1.4)
Wisbech	1296 (23.4%)	60.7 ± 11.9	1.0** (0.1; 1.9)	1.2* (0.3; 2.1)	0.8 (−0.0; 1.7)	1165 (23.4%)	64.0 ± 10.8	2.0* (1.2; 2.8)	1.9* (1.1; 2.7)	1.2* (0.4; 2.0)
Seasonality										
Spring			−0.1 (−0.5; 0.3)	−0.1 (−0.5; 0.3)	−0.1 (−0.5; 0.3)			−0.4 (−0.8; 0.0)	−0.3 (−0.7; 0.1)	−0.3 (−0.7; 0.1)
Winter			−1.4* (−1.8; −1.0)	−1.2* (−1.7; −0.8)	−1.2* (−1.6; −0.8)			−0.4 (−0.8; 0.0)	−0.1 (−0.5; 0.3)	−0.1 (−0.5; 0.3)
PAEE (kJ·d^−1^·kg^−1^)				0.1* (0.1; 0.1)	0.1* (0.1; 0.1)				0.1* (0.1; 0.1)	0.1* (0.1; 0.1)
BMI (kg·m^−2^)					0.4* (0.3; 0.4)					0.4* (0.4; 0.5)

The Fenland study 2005 to 2015.

Unadjusted values represent mean ± SD V̇O_2_max estimates computed within each substratum. Model values are mean (95% CI). Model 1 is mutually adjusted for sociodemographic characteristics. Model 2 is additionally adjusted for PAEE (kJ·d^−1^·kg^−1^). Model 3 is additionally adjusted for BMI (kg·m^−2^). Mean ± SD PAEE for women: 49 ± 20 kJ·d^−1^·kg^−1^; for men: 59 ± 23 kJ·d^−1^·kg^−1^. Mean ± SD BMI for women: 26.5 ± 5.3 kg·m^−2^; for men: 27.3 ± 4.1 kg·m^−2^.

*P < 0.01.

**P < 0.05.

Sequentially adjusted multivariable analyses of associations between estimated V̇O_2_max_tbm_ and sociodemographic characteristics are reported in Table 2. Age was still inversely associated with estimated V̇O_2_max_tbm_ but was attenuated to the null with adjustment for PAEE. Among women, occupation type and smoker status were not associated with differences in estimated V̇O_2_max_tbm_ after adjustment. Among men, estimated V̇O_2_max_tbm_ was higher in manual workers compared with sedentary workers when associations were adjusted for sociodemographic characteristics only. When additionally adjusted for PAEE and BMI, estimated V̇O_2_max_tbm_ was lower in manual workers (−0.9 mL O_2_^−1^·min·kg^−1^; 95% confidence interval [CI], −1.4 to −0.4), retirees (−1.4 mL O_2_·min^−1^·kg^−1^; 95% CI, −2.7 to −0.1), and the unemployed (−1.9 mL O_2_·min^−1^·kg^−1^; 95% CI, −3.6 to −0.3) relative to sedentary workers. Current male smokers had lower estimated V̇O_2_max_tbm_ relative to nonsmokers (−1.3 mL O_2_·min^−1^·kg^−1^; 95% CI, −1.9 to −0.7). In both women and men, estimated V̇O_2_max_tbm_ did not differ by education level, income, and marital status after adjustment. Table 3 presents analogous results for estimated V̇O_2_max_ffm_. The direction and magnitude of differences in estimated V̇O_2_max_ffm_ across sociodemographic factors were similar to those found for estimated V̇O_2_max_tbm_; however, Black men had lower V̇O_2_max_ffm_ (−7.1 mL O_2_·min^−1^·kg^−1^, 95% CI, −10.9 to −3.3) relative to White men.

**FIGURE 2:**
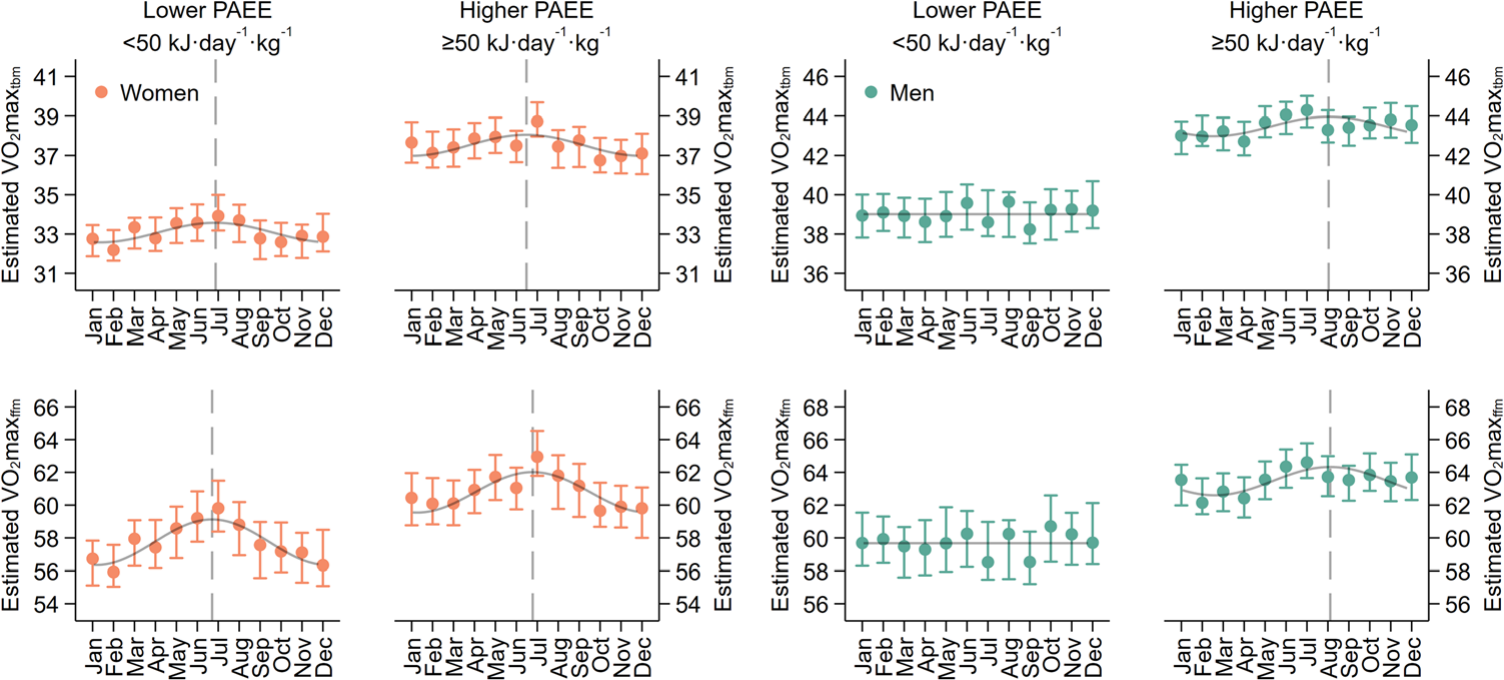
Seasonal variation in estimated maximal oxygen consumption per kilogram total body mass (V̇O_2_max_tbm_) and per kilogram fat-free mass (V̇O_2_max_ffm_) stratified by sex and PAEE levels. *Dots and bars* represent point estimates and 95% CIs from a binned regression procedure, adjusted for age, ethnicity, education level, work type, income, marital status, smoker status, and fitness testing site. Superimposed curves represent seasonal fitness values derived from an unadjusted cosinor model. *Vertical dashed lines* represent seasonal peaks in fitness values where seasonal variation is observed. The Fenland Study 2005 to 2015.

Women tested in the winter had lower estimated V̇O_2_max_tbm_ and V̇O_2_max_ffm_ than women tested at other times of the year. Seasonal variation in estimated V̇O_2_max_tbm_ and V̇O_2_max_ffm_ was not observed in men after adjustment for PAEE. To investigate this further, we analyzed seasonal variation in CRF_estimated_ when stratified by higher (≥50 kJ·d^−1^·kg^−1^) and lower (<50 kJ·d^−1^·kg^−1^) PAEE levels (Fig. 2). Seasonal variation in CRF_estimated_ persisted in men and women with higher PAEE levels. Women with lower PAEE levels also demonstrated seasonal variation, however CRF_estimated_ did not differ by season in men with lower PAEE.

## DISCUSSION

Here we have examined how CRF estimated from a submaximal exercise test (CRF_estimated_) varies by anthropometric, sociodemographic, and behavioral characteristics in a population-based sample of UK adults (The Fenland Study). The direct relationship between physical activity and CRF_estimated_ was stronger than the inverse relationship between age and CRF_estimated_. Although CRF and CRF trainability are known to have a strong genetic component (24), our findings reinforce the importance of maintaining an active lifestyle in adulthood to counteract declines in CRF with age. This extends previous reports of CRF in the United Kingdom and provides direction for future population-based studies of CRF.

CRF_estimated_ was lower in older versus younger UK adults, however the magnitude of difference per 5 yr (approximately 0.3 mL O_2_·kg^−1^·min^−1^) was less than values published in some CRF registries from different countries. Cardiorespiratory fitness declined by approximately 2 mL O_2_·kg^−1^·min^−1^ per 5 yr in the US-based FRIEND registry (25), Brazil-based Fleury study (26), and Norway-based HUNT study (27). Similarly, the age-gradient in the Prevention First Registry (28) was 1.5 mL O_2_·kg·min^−1^ but 1 mL O_2_·kg^−1^·min^−1^ in the SHIP study (29), both from Germany, whereas the Danish Health Examination Survey (30) and Korean-based KISS FitS study (31) reported a difference of 1.3 mL O_2_·kg^−1^·min^−1^ per 5 yr. Caution should be used when making direct comparisons across these studies because of differences in CRF measurement approaches, sampling of study populations, and different eras in which these studies were conducted.

Unadjusted CRF_estimated_ reported in the present study from the East of England were generally greater than values reported by previous UK-based population studies of CRF. In a sample of male factory workers assessed from 1979, Tuxworth et al. (7) found mean V̇O_2_max_tbm_ to be 29.9 mL O_2_·min^−1^·kg^−1^. In the Welsh Heart Health Survey from 1985 (6), mean V̇O_2_max_tbm_ among women 30 yr or older was 28.4 mL O_2_·min^−1^·kg^−1^ and among men was 33.7 mL O_2_·min·kg^−1^. In the Allied-Dunbar National Fitness Survey from 1990 (8), mean V̇O_2_max_tbm_ was 29.9 mL O_2_·min^−1^·kg^−1^ in women and 38.9 mL O_2_·min^−1^·kg^−1^ men. More recent studies of CRF in the UK (9,10) report values greater than these. It is possible that CRF in UK adults has improved since these earlier surveys due to the adoption of improved health behaviors in the general population. However, the difference in mean CRF values between these historical studies and the present study may be due to differences in study design. The Fenland cohort may have also recruited a higher proportion of healthy and enthusiastic people compared with the general population (11). Thus, it is possible that higher CRF_estimated_ values reported here reflect regional differences in health across the United Kingdom.

The association between CRF_estimated_ and physical activity was stronger than that for age; a one SD higher PAEE equates to the same difference in CRF (~2 mL O_2_·kg^−1^·min^−1^ higher) as being 25 yr younger. Similarly, a one SD higher BMI was associated with lower CRF (1.2 to 1.6 mL O_2_·kg^−1^·min^−1^), adjusted for age, physical activity and other sociodemographic characteristics. Thus, physical activity and BMI were stronger determinants of CRF than age and other factors that were examined. Physical activity is known to improve and explain a majority of the variance in CRF among adults (32–34), but age-related decline in CRF is not wholly due to physical inactivity (35,36). Reduced cardiac output and impaired skeletal muscle oxidative capacity with age are also contributing factors, particularly after the seventh decade of life (37–39). We did not directly measure cardiac output or skeletal muscle function, and therefore cannot investigate whether the preservation of CRF with age is related to maintenance of these factors. We also did not obtain CRF_estimated_ in adults 70 yr and older, when the impact of higher physical activity on improved CRF may wane. Nevertheless, our data suggest that higher physical activity can alter the trajectory of CRF decline with age in generally healthy adults. It is unclear whether this finding is reflective of health promotion strategies to increase physical activity and CRF within the Cambridgeshire region (40). If so, future work could seek how these strategies may be adapted to UK regions with high cardiometabolic disease prevalence.

People in more physically demanding occupations were fitter than those in sedentary occupations. When accounting for PAEE and BMI, however, CRF_estimated_ did not differ by occupation in women; in men, CRF_estimated_ was statistically lower in manual, retired, and unemployed workers compared to sedentary workers. Other studies report that manual workers may have greater muscle strength but lower CRF than the general population (41,42). This may be due to sedentary workers participating in leisure-time physical activity, which may have more CRF-enhancing effects than workplace physical activity. Although it is not immediately clear the mechanism by which manual work would lower CRF in men, it is likely occupation specific and could be related to diminished lung function from exposure to occupational respiratory health hazards (43). Previously, we reported that manual workers in the Fenland cohort had greater physical activity levels than other occupation types (11). It is therefore reassuring to observe that the negative effect of manual work on CRF—whatever the mechanism—is partly ameliorated by higher physical activity among male workers. Alternatively, the observed association between low CRF and manual work could be due to residual confounding for socioeconomic status. Previous research suggest that lower CRF in retired and unemployed male workers is related to advanced age and cardiovascular deconditioning after long-lasting physical inactivity (44).

We present CRF_estimated_ scaled by both total body mass (V̇O_2_max_tbm_) and fat-free mass (V̇O_2_max_ffm_). In multivariable analysis, estimated V̇O_2_max_tbm_ was negatively associated with BMI, however estimated V̇O_2_max_ffm_ was positively associated. Other studies demonstrate that CRF is independent of adiposity when scaled by fat-free mass and suggest V̇O_2_max_ffm_ can be considered an indirect measure of musculoskeletal tissue metabolic quality (45,46). More direct measurements of muscle oxidative capacity, such as tissue biopsy or imaging (47), could be used to elucidate whether this is preserved in otherwise overweight and obese participants with higher V̇O_2_max_ffm_ values. Indeed, ectopic fat infiltration of skeletal muscle may be more related to impaired muscle oxidative capacity and reduced force production than overall adiposity (48,49).

CRF_estimated_ values were generally higher in the summer compared to the spring and winter. Physical activity behaviors displayed a similar pattern as reported in previous analyses (11). PAEE adjustment negated seasonal variation of CRF in men, however in women seasonal variation persisted. Given the cross-sectional analysis used in this study, we recognize that the results regarding CRF seasonality should be interpreted cautiously and a repeated measures design would be more appropriate for quantifying seasonal variation. Such a design would be less feasible for a population-based study of CRF, however, since increased test frequency would be costly to scale and could increase lost-to-follow-up rates. Future work could elucidate whether seasonal variation in CRF among women is related to seasonal variation in endogenous factors, such as circannual hormonal rhythms (50).

Our study has strengths and limitations. We objectively assessed CRF_estimated_ in a large participant sample, enabling the investigation of differences by sociodemographic characteristics in the UK. We also quantified and compared the influence of objectively measured PAEE and BMI on associations between CRF_estimated_ and these characteristics, which allow judgment of their relative importance. A limitation of our study includes using heart rate response to a submaximal exercise test, rather than directly measured maximal oxygen consumption. We show in the validation substudy that the CRF_estimated_ from this method agree with direct measurements of CRF, which provides reassurance of our findings. Other sources of CRF estimation error do still exist, however, including the conversion of the energetic cost of the treadmill test to oxygen cost and the estimation of resting energy expenditure. This could impact the classification of participants into CRF categories in clinical settings (51). Another potential limitation is the non-representativeness of the Fenland cohort compared to the random population sampling frame. Compared to non-responders, participants were slightly older, leaner, less likely to smoke, more likely to drink alcohol, and less likely to live in deprived neighborhoods. These differences were small, however, and observed physical activity levels—the strongest determinant of CRF—were similar to those observed in the general UK population (11,52), suggesting findings may generalize more widely.

## CONCLUSIONS

We have described variation in CRF_estimated_ within a UK adult population by sociodemographic factors and lifestyle behaviors. CRF_estimated_ was inversely associated with age but less steeply than anticipated, suggesting older generations are comparatively fitter than younger generations. Physical activity and body size were stronger determinants of the variance in CRF_estimated_ than any other factor including age. A one SD difference in physical activity had the same impact on CRF as being 25 yr younger. This emphasizes the importance of maintaining physical activity across adulthood.
